# Development of *Streptococcus pneumoniae* Vaccines Using Live Vectors

**DOI:** 10.3390/vaccines2010049

**Published:** 2014-01-07

**Authors:** Shifeng Wang, Roy Curtiss

**Affiliations:** 1 Center for Infectious Diseases and Vaccinology, The Biodesign Institute, Arizona State University, Tempe, AZ 85287, USA; 2 School of Life Sciences, Arizona State University, Tempe, AZ 85287, USA; E-Mail: rcurtiss@asu.edu

**Keywords:** *Streptococcus pneumoniae*, *Salmonella*, lactic acid bacteria, BCG, adenoviruses, bacterial vector, viral vector

## Abstract

*Streptococcus pneumoniae* still causes severe morbidity and mortality worldwide, especially in young children and the elderly. Much effort has been dedicated to developing protein-based universal vaccines to conquer the current shortcomings of capsular vaccines and capsular conjugate vaccines, such as serotype replacement, limited coverage and high costs. A recombinant live vector vaccine delivering protective antigens is a promising way to achieve this goal. In this review, we discuss the researches using live recombinant vaccines, mainly live attenuated *Salmonella* and lactic acid bacteria, to deliver pneumococcal antigens. We also discuss both the limitations and the future of these vaccines.

## 1. Introduction

*Streptococcus pneumoniae* is the most common cause of pneumonia as well as a number of invasive diseases, such as meningitis and sepsis, and non-invasive mucosal diseases, such as otitis media and sinusitis. It causes severe morbidity and mortality worldwide, especially in young children and the elderly [1]. It has been estimated that 14.5 million episodes of serious pneumococcal disease occur each year, resulting in 826,000 deaths in children under 5 years of age, the vast majority of which occur in low-income countries with poor access to health care [1]. The overall rate of invasive pneumococcal disease (IPD) among children and adults is reported to be between 11 and 23.2/100,000 individuals per year [2]. In adults, community-acquired pneumonia, among which 30%–50% are caused by *S. pneumoniae* [2], is one of the major respiratory health diseases in the USA and Europe [2,3]; it is the most frequent cause of death from infection and poses a heavy burden to healthcare systems worldwide [2,4]. It is estimated that the annual total economic burden of pneumococcal disease among US adults aged over 50 years is about $5.5 billion [5]. The increasing incidence of antibiotic-resistant *S. pneumoniae* strains worldwide posed another threat to the treatment of infection [2]. The burden of pneumococcal diseases is worsened by increasing numbers of people with chronic disease (sickle-cell disease, chronic renal failure, chronic liver disease, asplenia), HIV or mycobacterial infection, as well as an aging population in many developed countries [2]. Currently, we have two types of vaccines against *S. pneumoniae*, pneumococcal polysaccharide vaccine (PPV) and pneumococcal conjugate vaccine (PCV) [6,7]. Both of them are designed to generate antibodies against capsular polysaccharide (CPS) [8,9]. *S. pneumoniae* has at least 94 serotypes with different abilities in nasopharyngeal carriage, invasiveness and disease incidence. The PPV is a 23-valent vaccine, which covers 23 commonly encountered serotypes. It is recommended to persons older than 65 years of age and aged ≥2 years at high-risk for pneumococcal diseases. The CPS is a T-cell-independent immunogen. It does not lead to isotype switching and induction of memory B-cell responses, leading to a temporary protection [10]. Capsular vaccines could cause hyporesponsiveness that blunts the immune response to subsequent doses due to the first dose [2]. It is also not very effective in infants and children under 2 years old—a group that is highly susceptible to infection, particularly in developing countries. Several meta-analyses showed that this vaccine is effective in low-risk adults, but not in high-risk groups [11,12,13]. To conquer these problems, the conjugate vaccine, PCV7, was licensed in 2000. Recently, PCV10 and PCV13 were licensed too. These vaccines are composed of pneumococcal polysaccharides conjugated to different protein carriers [6]. A conjugate vaccine can present the peptide or carbohydrate epitopes to carrier-peptide- or carbohydrate-specific T cells, respectively [14], resulting in T cell help for the production of memory B cells [10,15] and robust immune responses [16]. The conjugate vaccine increases enabling it to be used for young children [17]. The introduction of conjugate vaccines has tremendously decreased the rate of IPD and nasopharyngeal carriage by vaccine serotypes in children [15,18,19]. An effective herd immunity was also observed [7,20,21] with total 2/3 IPD reduction [6].

At the same time, some non-vaccine serotypes become prevalent in the face of the introduction of conjugate vaccines [22,23,24]. Also, certain high-risk groups have poor immunological responses to some of the polysaccharides in the vaccine formulations [25]. There are also concerns about the conjugate vaccines related to the high cost and complexity of manufacture due to the different prevalent serotypes in different geographical areas and the limited coverage of the current PCV vaccines [26]. Thus, to develop a low-cost, effective vaccine against *S. pneumoniae* is still urgent. The new vaccine should be able to induce more effective and durable immune responses that could potentially protect against all clinically relevant pneumococcal capsular types and cover some high-risk groups who may not respond well to the current vaccine, while keeping the cost low enough to be used in developing countries. The success of vaccines against other pathogens encourages the scientific community to develop a pneumococcal vaccine based on conserved protein antigens across all capsular types [26]. Different reviews have previously covered topics related to new generations of *S. pneumoniae* vaccines [6,27,28], animal models [29,30], antigen selection [26,31,32,33] and mechanisms of protection [28,34].

In this review, we will focus on developing new-generation pneumococcal vaccines using live bacteria delivering conserved protein antigens. We will discuss two types of live vaccines, live attenuated *Salmonella* and live lactic acid bacteria (LAB), mainly *Lactococcus* and *Lactobacilli*. Recombinant bacterial strains have several advantages. They have intrinsic adjuvant properties and can deliver antigens or DNA vectors with its native form using mucosal routes, which mimic the natural infection process to induce immune responses against the heterologous antigens in both mucosal and systemic sites. The productions of these kinds of vaccines are easier and less expensive than that of protein-based subunit vaccines. Several of the best-characterized candidate *S. pneumoniae* antigens, including pneumococcal surface protein A (PspA), pneumococcal surface adhesin A (PsaA), pneumococcal surface protein C (PspC), and pneumolysin (Ply), have been tested in various live vectors including attenuated pathogenic bacteria and nonpathogenic bacteria (Table 1). We will focus on the immune responses induced by these recombinant bacterial vaccines. The detailed properties of different protein antigens tested in live vaccines have been discussed elsewhere [32].

## 2. Bacterial Vectors Deliver Protective Antigens

### 2.1. Salmonella-Vectored Vaccines

*Salmonella* is a pathogenic bacterium. In order to be used as a live vaccine vector, it should be attenuated by various mutations [35,36]. Furthermore, multiple mutations are introduced to reduce the chance of reverting to display virulence. *Salmonella* is one of the most widely studied live vectors to deliver protective antigens. Recombinant attenuated *Salmonella* vaccines (RASVs) can attach to, invade and colonize in deep effector lymphoid tissues after mucosal delivery and therefore remodel the host cells that they target as well as promote immunomodulatory effects to induce immune responses in locations where bacteria persist as well as at systemic sites [37,38,39]. Currently, a phase I clinical trial showed that the three *S. typhi* vaccine vectors—χ9633, χ9639 and χ9640—delivering pneumococcal antigen PspA were safe and well-tolerated [40]. These achievements were made during the process with a final goal of developing a safe RASV suitable for use in newborns/neonates and infants that induces protective immunity to the diversity of *S. pneumoniae* strains. 

**Table 1:** Live vectored vaccines for *S. pneumoniae.*

Strain ^1^	Antigen	Antigen source ^2^	Promoter/secretion signal	Location	Mice ^3^/rabbit	Schedule	Route/dose ^4^	Immune responses ^5,6^	Challenge Strain/Route/Dose ^7^	Protection	Ref.
*Bacterial vectors*											
*S. typhimurium*											
*Single antigen*											
C5 *aroA*	Ply (Pd-B, W433F)	Type 1 Ply overexpression strain	Native promoter	Cytoplasm	BALB/c	Day 0, 14, 28, 42	Oral 10^10^	Serum IgG, IgA	N.A	N.A	[41]
					Quackenbush mice Outbreed	Day 0, 14,28	i.p. 10^6^	Serum Ig G IgA	N.A	N.A	
χ9101, χ9017, χ9241	PsaA	Tigr 4 (4)	P_trc_, native SS, *lpp*, *bla*, *Y.pestis psaA* SS	Periplasm	BALB/c, C57BL/6	Day 0, 42	Oral 10^9^ i.n. 10^9^	Serum IgG, Vaginal wash, nasal and lung IgA	WU2 (3), i.p. 2 × 10^4^; L82016 (6B), E134 (23), i.n. 5 × 10^6^; A66.1(3), D39 (2), i.n. 1 × 10^7^	i.p. no protection; i.n. reduce nasal colonization, but not lung	[42]
SR-11 χ4550	PspA Rx1 (aa 1–470)	Rx1	P_trc_, native signal	Periplasm and cytoplasm	BALB/cJ, CBA/N *xid*	Day 0, 56, 140	Oral 1.5 × 10^9^	Serum, VL, IL IgG, IgA, IgM, spleen, PP, PBMC PspA-specific IgG IgM IgA APC	WU2 (3), i.p. 3 × 10^3^, i.v. 10^4^	66% protection, passive protection by serum: i.v. 33%–89%; i.p. 43%	[43]
					New Zealand White rabbits	Day 0, 30	Oral 1.6 × 10^10^	Serum, VL IgG			[43]
SL1344 χ8501	PspA (aa 3–257)	Rx1	P_trc_, *bla* SS	Periplasm/Supernatant	BALB/c	Day 0 or Day 0, 70	Oral, 1.3–1.9 × 10^9^	Serum IgG, VL IgA	WU2 (3), i.p. 4.8 × 10^3^	60% Protection	[44]
SL1344 χ8501	PspA (aa 3–257)	Rx1	P_trc_, no signal or *bla* SS	Cytoplasm periplasm	BALB/c	Day 0	Oral, 10^9^	Serum IgG,	NA	NA	[45]
χ8937	PspA (aa 3–257)	Rx1	P_trc_, *bla* SS	Periplasm and lysed	BALB/c	Day 0, 7	Oral 1.3 × 10^9^ Boost 1.2 × 10^9^	Seum IgG, VL IgA	NA	NA	[46]
χ9241, χ9277, χ9373, χ9402	PspA (3–285)	Rx1	P_trc_, *bla* SS	Periplasm	BALB/c	Day 0	Oral 1 × 10^9^	Serum IgG, VL IgA. IL-4, IFN-γ ELISPOT, CD4, CD8, cytokines, memory T cell	WU2 (3), i.p. 2 × 10^4^	46%–47% for strains with Δ*sopB* mutation	[47]
χ9241	PspA 3–285	Rx1	P_trc_, *bla* SS+CT	Periplasm	BALB/c and C57BL/6, BALB/c pIgR^−/−^ , BALB/c and C57BL/6 MyD88^−/−^, MyD88^−/−^TRIF^−/−^	Day 0, 14, 28 or Day 0, 14	i.g. 10^9^, i.n. 10^8^	Serum, fecal IgG, IgA, Ag-specific CD4+ T cell proliferation, adoptive transfer	WU2 (3) i.v. 2 × 10^6^ or 2 × 10^7^, i.t. 5 × 10^7^	0% in MyD88^−/−^ mice	[48]
χ8133, χ9088, χ9558	PspA (3–257)	Rx1	P_trc_, *bla* SS	Periplasm	BALB/c	Day 0, 56	Oral 1 × 10^9^	Serum IgG, VL IgA. IL-4, IFN-γ ELISPOT, cytokines, Passive transfer of cells and sera	WU2 (3), i.p. 5 × 10^4^	21%, 86%, 71% for χ8133, χ9088 and χ9558; passive protection by serum 80%, 100%, 100%; by spleen cells, 0%, 100%, 60%	[49]
χ9241	PspA (aa 3–285) PspC (aa 4–404)	Rx1 L81905 (4)	P_trc_, *bla* SS, *bla* SS+CT, *phoA*, *ompA*	Periplasm	BALB/c	Day 0	Oral 10^9^	Serum IgG, VL IgA, ELISPOT IL-4, IFN-γ for PspA or PspC	WU2 (3), i.p. 2 × 10^4^ for PspA D39 (2), i.p. 4 × 10^3^ for PspC	*bla* SS–PspA, 63%; *bla* SS+CT–PspC 60%	[50]
χ9241, χ9852, χ9884	PspA 3–285	Rx1	P_trc_, *bla* SS	Periplasm	BALB/c	Day 0, 28	Oral 10^9^	Serum IgG, VL IgA	WU2 (3). i.p. 4 × 10^4^	23%–45% for different *rfaH* mutations	[51]
χ9558	PspA 3–285	Rx1	P_trc_, *bla* SS	Periplasm	BALB/c Neonatal (7-day-old) and infant (21-day-old)	Day 0, 21, 42	Oral, i.n. 5 × 10^8^	Serum IgG , VL IgA . IL-4, IFN-γ ELISPOT	WU2 (3). i.p. 2 × 10^3^ for oral route, 4 × 10^3^ for i.n.	baby mice from immunized mother 40% (7 day) or 50% (21 day); from naïve mother 11% (7 day) or 10% (21 day)	[52]
χ9241, χ9853, χ9885	PspA 3–285	Rx1	P_trc_, *bla* SS	Periplasm	BALB/c	Day 0, 28	Oral 10^9^	Serum IgG, VL IgA	WU2 (3). i.p. 4 × 10^4^	55%–77% for different *rfc* mutations	[53]
χ9095, χ9241, χ9555, χ9959	PspA 3–285	Rx1	P_trc_, *bla* SS	Periplasm	BALB/c	Day 0, 42	Oral 10^9^	Serum IgG, VL IgA	WU2 (3). i.p. 2 × 10^4^	52% for *relA198*, 39% for *relA 197*	[54]
χ9241, χ9281	PspA 3–285	Rx1	P_trc_, *bla* SS, *bla S* +CT	Periplasm	BALB/c	Day 0, 7, 14, 21	i.n. OMV contain 350 ng PspA from *Salmonella*	Serum IgG, VL IgA	WU2 (3). i.p. 0.246–4 × 104	100% protection against low dose, 47% for high dose	[55]
χ 9241, χ9555	PspA 3–285	Rx1	P_pagC_, P_ssaG_, P_trc_ *bla* SS	Periplasm	BALB/c	Day 0, 42	Oral 10^9^	Serum IgG, VL IgA, IL-4, IFN-γ ELISPOT	WU2 (3). i.p. 2 × 10^4^	46% for regulated delayed antigen synthesis system, 39% for P_pagC_	[56]
χ9241, χ9844, χ9845, χ9846, χ9881	PspA 3–285	Rx1	P_trc_, *bla* SS	Periplasm	BALB/c	Day 0, 35	Oral 10^9^	Serum IgG, VL IgA	WU2 (3). i.p. 4 × 10^4^	1-dephosphorylated lipid A modifications do not affect protection	[57]
χ9241, χ9884, χ9885, χ11313, χ11314, χ11315, χ11316, χ11317	PspA 3–285	Rx1	P_trc_, *bla* SS	Periplasm	BALB/c	Day 0, 35	Oral 10^9^; i.n. 10^7^; i.p. 10^6^	Serum IgG, VL IgA	NA	NA	[58]
χ9241, χ9278, χ9848, χ9850, χ11318, χ11088	PspA 3-285	Rx1	P_trc_, *bla* SS	Periplasm	BALB/c	Day 0, 35	Oral 10^9^	Serum IgG, VL IgA	WU2 (3). i.p. 2 × 10^4^	23%–37%, not affected by palmitoylation state of lipid A	[59]
χ9241, χ11088, χ11089, χ11090, χ11091	PspA 3-285	Rx1	P_trc_, *bla* SS	Periplasm	BALB/c	Day 0, 35	Oral 10^9^	Serum IgG, VL IgA	WU2 (3). i.p. 2 × 10^4^	23%–31%, not affected by phosphate groups of Lipid A	[60]
χ9241	PspA 3-285	Rx1	P_trc_, *bla* SS+CT	Periplasm	C57BL/6, Ccr2^−/−^	Day 0	i.g. 10^9^	Serum IgG, IgA, BALF IgA	WU2 (3). i.t. 10^4^	long-term protection 80%; protect against 2nd pneumococcal pneumonia 80%–100%; Passive serum protection, 90%	[61]
Multiple antigen											
χ9241	PspA Rx1 (aa 3–285) EF5668 (aa 4–417)	Fusion of Rx1 and EF5668 (4)	P_trc_, *bla* SS	Periplasm	BALB/c	Day 1, 7, 42	Oral 10^9^	Serum IgG, VL IgA, complement deposition	WU2 (3), i.p. 2 × 10^4^; 3JYP2670 (3), i.v. 1 × 10^6^; A66.1 (3), i.n. 1 × 10^8^	Rx1-EF5668 83%–100%; Rx1, 33%–53%; EF5668 26%–66%	[62]
χ9760, χ9828, χ11018, χ11026	PspA PspC	Rx1 L81905(4)	P_trc_, P_lpp-lacO_, *bla* SS	Periplasm	BALB/c	Day 0,42	Oral 10^9^	Serum IgG	WU2 (3), i.p. 2 × 10^4^; L81905 (4), i.v. 1 ×10^6^; A66.1 (3), i.n. 1 × 10^8^	Dual-plasmid i.p. 75%; i.v. 100%; i.n. 80%	[63]
DNA vaccine											
χ4550	PspA PsaA	R6 (ATCC-255)	P_CMV_	Cytoplasm	BALB/c	Day 0, 35	Oral 1.5 × 10^9^	Serum, NL IgG, IgA	D39 (2), i.n. 10^6^	PsaA + PspA is best in reducing nasal colonization	[64]
*S. typhi*											
ISP1820, χ9633, Ty2, χ9639, χ9640	PspA 3–285	Rx1	P_trc_, *bla* SS	Periplasm	BALB/c	Day 0, 42	i.n. 1 ± 0.2 × 10^9^	Serum IgG , VL IgA, IL-4, IFN-γ ELISPOT	WU2 (3), i.p. 1 × 10^4^	50% for χ9633, 75% for χ9639; 81% for χ9640	[65]
ISP1820, χ9633, Ty2, χ9639, χ9640	PspA 3–285	Rx1	P_trc_, *bla* SS	Periplasm	Neonatal(7 day) and Infant (21 day) BALB/c	Day 0, 14, 28, 42	i.n. 5 × 10^8^	Serum IgG , VL IgA, IL-4, IFN-γ ELISPOT	WU2 (3), i.p. 4 × 10^3^	neonatal mice 33%–65%; infant mice 40%–75%	[66]
ISP1820, χ9633, Ty2, χ9639, χ9640	PspA 3–285	Rx1	P_trc_, *bla* SS	Periplasm	Adult human	Day 0	Oral 10^7^, 10^8^, 10^9^, 10^10^	ELISPOT IgA, serum IgA, IgG	N.A	Induce limited IgA response	[40]
*Lactococci* and *Lactobacilli*											
*Protein antigen*											
*L. casei* CECT5275 *L. plantarum* NCDO1193 *L. helveticus* ATCC 15009 *L. lactis* MG1363	PsaA	472/96 (6B)	lactococcal P1 promoter, *usp45* SS	Cell wall	C57Bl/6	Day 0, 1, 14, 15, 28, 29	i.n. 10^9^	Saliva, NL, BAL IgA Serum IgG	ATCC0603 (6B), i.n. 5 × 10^6^	Reduce nasal colonization only in *L. helveticus*-PsaA	[67]
*L. lactis* F17847	PspA aa 1–418	Tigr4 (4)	P_nis_	Cytoplasm	CBA/ca	Day 0, 21, 42	i.n. 10^9^	Serum and LL IgG, LL IgA	Tigr4 (4), i.p. 2 × 10^5^, i.n. 1–2 × 10^6^,	i.p.: LAB vaccine40%, protein 15%–20%; extend survival time against i.n. challenge	[68]
*L.* casei CECT5275	PspA (clade 1)	435/96 (14)	constitute P1 promoter	Cytoplasm	C57Bl/6	Day 0, 1, 14, 15, 28, 29	i.n. 10^9^	Serum IgG, NO Saliva, NL IgA; complement deposition assay	A66.1(3), i.p. 10^2^, heterologous challenge	33%	[69]
*L. casei* CECT5275	PspA (clade 5) PspC	122/02 (23F) 491/00 (6B)	constitute P1 promoter	Cytoplasm	C57BL/6	Days 0, 1, 14, 15, 28, 29	i.n. 10^9^	Serum, VW, BAL IgG, IgA, cytokines	ATCC 6303 (3), i.n. 10^5^	PspA 40%–60%; PspC 12.5%–20%	[70]
*L. casei* CECT5275	PspC	491/00 (6B)	constitute P1 promoter, *w/wo usp45* SS	Cell wall or cytoplasm	C57BL/6	Day 0, 1, 14, 15, 28, 29; Day 0, 14, 28 or Day 0, 1, 14, 15,	i.n. 10^9^ sublingual. 10^9^ prime-boost	No IgG and IgA in nasal sublingual	ATCC 0603(6B), i.n. 5 × 10^6^	Reduce nasal colonization by i.n. immunization, antibody primed after challenge	[71]
*L. lactis* NZ9000	PppA	T14 (14)	P_nisA_, usp45 SS	Cell wall	3 weeks (young) and 6 weeks (adult) Swiss Albino mice	Day 0, 14,28	i.n. 10^8^	Serum BAL IgM, IgG, IgA	T14(14), i.p. 10^8^, AV3(3), AV6(6B), AV14(14), AV23(23F), i.n. 10^6^	T14, i.p. 60%–70%; passive protection 40%–50%; i.n. reduce lung colonization of CPS type 3, 6B, 14, and 23F	[72]
*L. lactis* NZ9000	PppA	T14 (14)	P_nisA_, *usp45* SS	Cell wall	Male Swiss Albino mice	Day 0, 1, 2, 3, 4, boost at 2 weeks interval	Oral, 10^8^	Serum, BAL, IF-4 IgM, IgG, IgA, Opsonophagocytosis , Spleen IL-4, IFN-γ	T14(14) , AV3(3) , AV6(6B), AV14(14), AV23(23F), i.n. 10^6^	Reduce lung colonization of CPS type 3, 6B, 14, and 23F	[73]
*L. lactis* NZ9000	PppA	T14 (14)	P_nisA_, *usp45* SS	Cell wall	3 weeks, Swiss albino mice	Days 0, 14, 28	i.n. 108	Serum, BAL IgA, IgG , BAL cytokine	CPS type 3, type 14, i.n. 10^6^	With probotic, reduce lung and blood colonization of CPS type 3 and 14	[74]
*L. lactis* NZ9000	PppA	T14 (14)	P_nisA_, *usp45* SS	Cell wall	3 weeks, Male Swiss albino mice	Day 0, 1, 2, 3, 4, boost at 2 weeks interval	Oral 10^8^	Serum, IgM IgG; BAL, IgM, IgG, IgA; IF, IgA , Opsonophagocytosis , cytokines	AV3(3), AV6(6B), AV14(14), AV23(23F), i.n. 10^6^	prevent bacteremia of CPS types 6B, 14, and 23F, decreased lung colonization of CPS type 3	[75]
*Capsular polysaccharide antigen*											
*L. lactis* MG1363	Type 3 capsular polysaccharide	WU2 (3)	natural promoter	Mainly associated with cells	BALB/c	Day 0 and 49	i.p. 3.5 × 10^6^	serum IgM, IgG, IgG1 and IgG3	N.A.	N.A.	[76]
*L. lactis* NZ9000	Type 14 capsular polysaccharide	N.A.	natural promoters for structure gens and P_nisA_ for regulatory gene	Mainly supernatant	N.A.	N.A.	N.A.	N.A.	N.A.	N.A.	[77]
*Bacillus* Calmette-Guérin											
BCG	PspA	Rx1	P_hsp60_, natural SS signal, *mtb19* lipoprotein SS	Cytoplasm, secreted, membrane associate	BALB/c C3H/HeJ	Day 0 and 119	i.p. 10^6^	Serum IgG, passive protection	WU2 (3), i.p. 10^4^	Secret, membrane associated protein, 50%–80% in C3H mice, 70%–100% in BALB/c mice, passive protection by serum:100% protect PspA clade 1, 13, 24, 0% for clade 18	[78]
Viral vector											
Adenovirus	PsaA PspA PdB	D39 (2)	P_CMV_	N.A.	BALB/C	Day 0, 28, 56	i.n. 3 × 10^7^	IgG	D39 (2), i.n. 10^7^	Combination of two or three rAds reduce lung colonization	[79]

1. All *Salmonella* strains are derived from the UK-1 strain, unless otherwise specified; see references for detailed genotypes of strains; 2. The capsular polysaccharide (CPS) type is shown in parenthesis; 3. Mice are 5–8 weeks old, unless otherwise specified; 4. i.g., intragastric; i.n., intranasal; i.p., intraperitoneal; i.t., intratracheal, i.v., intravenous; 5.BAL: bronchoalveolar lavage fluid; IL, intestinal lavage fluid; VL, vaginal lavage fluid; LL, lung lavage fluid; NL, nasal lavage fluid; 6. ELISPOT (Enzyme-linked immunosorbent spot );7. N.A.: not available.

#### 2.1.1. *S. typhimurium* Delivers Single Protective Antigen

Several antigens, PsaA, PspA, PspC and Ply, delivered by different recombinant attenuated *S. typhimurium* vectors were tested in mice (Table 1). These efforts were dedicated to screening optimal secreting signals [50], antigen coding regions [42], combinations of antigens [62,63], antigen delivery methods [50,62,63], *Salmonella* genotype for increased immunity and safety [57,58,59,60,80] and concept for testing new technology platforms for *Salmonella* vaccines [54,56].

##### 2.1.1.1. Ply

Ply has activities of cytotoxic/cytolytic and complement activation to facilitate the growth and invasion of *S. pneumoniae* in lungs during the early stage of infection [81]. It is conserved in both amino acid sequence and antigenicity among clinical isolates [32]. Paton *et al*. used a *S. typhimurium* C5 *aroA* strain to deliver a detoxified Ply-PdB (W433F) by intraperitoneal (i.p.) and oral routes [41]. Both immunization routes resulted in significant IgG responses against Ply. However, only the oral route resulted in IgA responses. Until now, there is no animal protection study with Ply delivered by *Salmonella* vectors. 

##### 2.1.1.2. PsaA

PsaA is a conserved surface protein present in all 90 *S. pneumoniae* CPS groups [82,83,84]. PCR-restriction fragment length polymorphism analysis of 80 serotypes demonstrated the conservation of the gene using ten different enzymes throughout the entire length of the gene [84]. Monoclonal antibody studies showed that PsaA is present in 89 serotypes of *S. pneumoniae* [83]. *S. typhimurium* vaccine vector delivers PsaA in two ways, one as a prokaryotic synthesized antigen [42], another as a eukaryotic synthesized antigen by delivery of a DNA vaccine (see Section 2.1.3) [64]. *Salmonella* strain χ9241 encoding full-length PsaA induced significantly high titers of anti-PsaA IgG in serum and IgA in vaginal washes, nasal washes, and lung homogenates [42]. Although the gene was cloned from the CPS type 4 *S. pneumoniae* Tigr4 strain, *Salmonella*-PsaA vaccine reduced nasopharyngeal colonization by L82016 (type 6B CPS) and E134 (type 23 CPS) in two strains of mice, BALB/c (haplotype H2^d^) and C57BL/6 (haplotype H2^b^), independent of whether mice were immunized by the oral or intranasal (i.n.) route. However, immunization could not reduce lung colonization by pneumococcal strains A66.1 (type 3 CPS) and D39 (type 2 CPS). The *Salmonella*-PsaA vaccine conferred no protection against i.p. challenge with *S. pneumoniae* strain WU2 (type 3 CPS). The work also indicated that the full length of PsaA with its native conformation might be important to induce protective immunity, which is different from PspA, in which the α-helical segment is enough to induce protective immune responses [85]. 

##### 2.1.1.3. PspA

Different from PsaA, PspA is a serologically variable surface protein. It is classified into three families and subdivided into six clades based on the *C*-terminal 100 amino acids of the α-helical region [86]. PspA family 1 is composed of clades 1 and 2, family 2 is composed of clades 3, 4 and 5. Family 1 and 2 PspA cover over 95% of clinical isolates typed to date [86,87]. Their distribution remains unaltered following the introduction of the PCV7 [88]. Although PspA has variability, different PspAs are cross-protective against different *S. pneumoniae* strains expressing different CPSes and serologically divergent PspAs [89,90]. PspA, delivered by *Salmonella* vectors, was tested by different groups using either prokaryotic expression vectors or eukaryotic DNA vectors in mice (Table 1). 

In our group, Nayak *et al*. first reported that PspA Rx1 (aa 1–470) with its native secretion signal encoded on a high copy plasmid is delivered by a *S. typhimurium* SR-11 Δ*cya* Δ*crp* strain [43]. This construction can induce significant anti-PspA IgG, IgA and IgM antibody titers in sera, vaginal washes and intestinal washes in both *Salmonella-*susceptible BALB/cJ mice (haplotype H2^d^) and *Salmonella*-resistant CBA/N xid mice (haplotype H2^k^) [43]. Enzyme-linked immunosorbent spot (ELISPOT) analyses showed anti-PspA IgG, IgM and IgA ASCs (antigen-secreting cells) in spleen, peripheral blood and Peyer’s patches. The anti-PspA IgG and IgA can also be detected in rabbit serum and vaginal washes following oral immunization with the RASV strain. Mice immunized with *Salmonella*-PspA were protected against i.p. challenge with the virulent WU2 strain. The passive protection experiments showed that the immune serum can protect mice against intravenous (i.v.) (CBA/N mice) and i.p. challenge (BALB/c mice) with the WU2 strain. This work set the foundation for the following studies [44,50].

Kang *et al.* replaced the native secretion signal for the PspA Rx1 with the β-lactamase secretion signal [44]. The fusion protein encoded amino acids 3–257 of PspA on a medium copy plasmid with a reduced synthesis level of the Asd selection marker [44]. A new *Salmonella* SL1344 vaccine strain χ8501 (Δ*crp-28* Δ*asdA16*), delivering this novel plasmid pYA3494, induced serum IgG and vaginal IgA against PspA and conferred protection against i.p. challenge with the WU2 strain. Notably, with only one-time immunization, the IgG response induced by χ8501(pYA3494) against PspA was higher than that against LPS or OMPs, both are indications of the immunogenic potential of the bacteria. This was also observed in a RASV strain with a regulated programmed lysis system vectoring the same *bla* SS-PspA fusion protein (Table 1) [46]. Further experiments showed that *Salmonella* vaccine strains delivering secretory proteins induced higher antibody responses than a strain delivering cytoplasmic PspA protein [45]. These data showed that antigen location in RASVs is important to induce antibody responses following oral immunization. 

Xin *et al.* further evaluated the effects of different Type II signal sequences (SS), including the *N*-terminal signal sequence of β-lactamase (*bla* SS), *N*- and *C*-terminal sequence of β-lactamase (*bla* SS+CT), *ompA* SS and *phoA* SS, fused with the α-helical domain of PspA Rx1 (encoding PspA Rx1 amino acids 3–285) on the immune responses [50]. The results showed that the strain carrying plasmid pYA4088 with *bla* SS–PspA fusion yielded the largest amounts of secreted PspA than other signals. Mice immunized with this construction developed the highest serum IgG and vaginal IgA antibody levels, IL-4 and IFN-γ secretion. Immunized mice were protected against i.p. challenge with a virulent *S. pneumoniae* strain. Thus, the PspA production level in RASVs is important for the protection against *S. pneumoniae* challenge [50]. 

The protection conferred by different *Salmonella* strains vectoring plasmid pYA4088 has been documented through a series of studies (Table 1). The plasmid was used to test our innovation technologies, regulated delayed *in vivo* antigen synthesis strategy, and regulated delayed attenuation [54,56,91], as well as in a phase I clinical trial [40]. It was used to further explore the effects of different mutations, such as lipid A and O antigen, on the safety and immunogenicity of *Salmonella* vaccine vectors [51,53,55,57,58,59,60] (Table 1). These tested mutations either directly contribute towards the construction of *S. typhimurium* vaccine strain χ9558 and *S. typhi* vaccine strains χ9633, χ9639 and χ9640 with the same genotype or can be used to further improve *S. typhimurium* or *S. typhi* vaccine vectors [80]. 

Strain χ9558 is the representative of a new generation of RASVs displaying wild-type characteristics at the time of immunization and becoming attenuated after colonization of host tissues. It exhibits an improved safety profile in adult mice, with a reduced ability to cause meningitis when administered orally, i.n., or i.p. [92]. It is totally safe and noninflammatory in newborn mice at doses equal to 10^7^ times the 50% lethal dose of the wild-type parent [91]. In adult mice, the strain χ9558, carrying a *pspA* expression plasmid, induces significantly higher levels of anti-PspA serum IgG and mucosal IgA antibodies than χ8133, a vaccine strain generated by a traditional way. Splenic lymphocytes from mice immunized with χ9558 produce significantly more IL-4 and IFN-γ secretion cells than that from mice immunized with χ8133, as well as increased levels of both Th1-specific cytokines (IL-2, IL-12, TNF-α) and Th2-specific cytokines (IL-4, IL-5, IL-10). Vaccination with χ9558 confers a greater degree of protection against *S. pneumoniae* challenge than that with χ8133 (71% *vs*. 21% survival, *p* < 0.01) [49]. Strain χ9558(pYA4088) is also immunogenic in infant and neonatal mice born from naïve or immunized mothers when administrated orally or i.n. and induce protective immunity against *S. pneumoniae* challenge [52].

Another plasmid pYA3802 with *bla* SS-PspA-CT (PspA aa 3–285) was used to probe the protective immune mechanisms of RASVs via the oral route. Park *et al*. proved that the sIgA is important to RASV-PspA-induced protection against intratracheal (i.t.) challenge using pIgR^−/−^ mice which lack the IgA secretion pathway [48]. Peyer’s patch plays an indispensable role for induction of PspA-specific IgA in both systemic and mucosal compartments. MyD88-mediated innate immunity is not essential for induction of Ag-specific B-cell responses induced by RASV synthesizing T-cell-dependent exogenous Ag, but it is critical for the protection against virulent *S. pneumoniae* challenge. Influenza infection followed by pneumococcal infection can cause severe pneumonia and this secondary pneumococcal pneumonia is the most common cause of influenza-associated death. Seo *et al.* tested whether the vaccine against S. pneumoniae could reduce the disease burden caused by seasonal epidemic and pandemic influenza [61]. Mice vaccinated orally with a RASV strain carrying plasmid pYA3802 resulted in attenuated pulmonary inflammation and effective long-term protection against secondary pneumococcal pneumonia after influenza infection [61]. Thus, oral RASV-PspA immunization is not only an efficacious way to protect against respiratory bacterial pathogens, but is also a promising approach against the impact of annual epidemic and pandemic influenza outbreaks. These results highlight the importance of immunizing both the young and elderly populations, which are more susceptible to infection by both *S. pneumoniae* and influenza, with a RASV against *S. pneumoniae*.

##### 2.1.1.4. PspC

PspC is another candidate surface antigen [93,94]. It plays an important role in the virulence of *S. pneumoniae* and protects mice against pneumococcal challenge in carriage [95] and sepsis models [94,96]. Xin *et al*. evaluated PspC from *S. pneumoniae* strain L82015 fused with different secretion signals as mentioned above [50]. The induced immune responses varied depending on the signal sequence used. Strains carrying the *bla* SS-PspC-CT fusions yielded the largest amounts of secreted PspC, induced the highest serum IgG and vaginal IgA titers, highest IL-4 and IFN-γ responses, and conferred the greatest protection against virulent *S. pneumoniae* i.p. challenge than other signal sequences fused to PspC. These results are consistent with the PspA results, which demonstrate that the antigen synthesis levels in live bacterial vectors are critical for induction of protective immune responses against *S. pneumoniae.* Using LAB as vectors delivering PsaA also confirmed this conclusion [67]. 

#### 2.1.2. *S. typhimurium* Delivers Multiple Antigens

To develop an effective vaccine against *S. pneumoniae*, multiple antigens are preferred to set blockages during the stages that *S. pneumoniae* attaches to, invades into and spreads in the host. *Salmonella* has the capacities to deliver multiple antigens with various approaches: (1) as fusion antigens on one plasmid delivered by one strain [62]; (2) as individual antigens on one vector delivered by one strain; (3) as individual antigens on different vectors delivered by one strain [63]; and (4) as a mixture of multiple strains, each specifying individual antigens [63]. These approaches require optimization of each component. 

We first tested if a RASV strain delivering one vector encoding PspA fusions could induce protections against multiple *S. pneumoniae* strains [62]. PspA is grouped into three families due to its diversity [86]. It is necessary to use PspAs from different families to elicit effective cross-protective coverage. Previously, we described the use of PspA from the Rx1 strain, which is from family 1. We chose another PspA from strain EF5668 from family 2. We also included the proline-rich domain of EF5668, which has been shown to encode protective epitopes that cross-protect against a variety of *S. pneumoniae* strains [94,97]. We evaluated fusion constructions consisting of PspA Rx1 and EF5668 with different orders in one vector to screen the best combination for an anti-pneumococcal vaccine. Both fusions elicited serum IgG and mucosal IgA to both families of PspA and strongly augmented percentage of cells with surface-bounded C3 on strains expressing family 1 and 2 PspAs. One of the fusion constructions, Rx1-EF5668, extended and enhanced protection against multiple strains of *S. pneumoniae* by i.p., i.v., or i.n. challenge [62]. This fusion construction of antigens from different families represents an important strategy for *S. pneumoniae* vaccine development. 

We then evaluated the way to deliver multiple antigen genes in separate vectors in the case that a fusion construct of multiple protective antigens is not the optimal choice when a multivalent vaccine is desired. The major challenge to achieve this goal is that the recombinant vaccine strain should stably maintain two or more expression vectors simultaneously, each carrying a unique selectable marker. To facilitate this strategy, we used a DadB^+^ vector to deliver the *pspC* gene, together with an Asd^+^ plasmid carrying the *pspA* gene to form a dual-plasmid system, which could deliver multiple antigens in a vaccine strain with Δ*alr* Δ*dadB* and Δ*asd* mutations [63]. The DadB^+^ plasmids are compatible with Asd^+^ vectors in a single vaccine strain without comprising the synthesis of individual antigens. Both plasmids are stable over 50 generations of growth, suggesting that antigen synthesis and delivery *in vivo* are not compromised in this system [63]. To further reduce the possible recombination between plasmids, a *recF* mutation was introduced into strains [63]. The *Salmonella* vaccine strain carrying both PspA and PspC by Asd^+^ and DadB^+^ vectors, respectively, induced higher serum and secretory antibody responses than the strain delivering a single antigen or a mixture of two vaccine strains each specifying one protective antigen and offered superior protection against i.p., i.v., or i.n. challenge with different serotypes of *S. pneumoniae* [63]. The DadB^+^-Asd^+^ dual-plasmid system represents another important tool to develop multivalent live recombinant vaccines [63].

#### 2.1.3. *S. typhimurium* Delivers DNA Vaccine

DNA vaccines encoding *psaA* and *pspA* have been shown to be effective in inducing antibody responses and Th1 immunity [98], which are important against pneumococcal infection [98,99,100]. However, preparation and characterization of DNA vaccines need complex procedures [101]. These procedures increase the cost of final products. DNA vaccines also induce poor mucosal responses in the nasopharynx. Zhang *et al.* used *Salmonella* to deliver multi-antigen-encoding DNA vaccines encoding *psaA* and *pspA* genes [64]. They modified the DNA vector by replacing the selection marker from ampicillin to Asd to better maintain the vector and reduce the safety concern due to the use of antibiotic selection markers. They also eliminated the neomycin-resistance selection marker for the same concern. The modified vector was used to clone *psaA* and *pspA* genes. *Salmonella* delivering DNA vaccines encoding *pspA* or *psaA*, either alone or mixed together, significantly reduced *S. pneumoniae* colonization in nasal washes compared with control. Mice orally immunized with RASV carrying multi-antigen DNA vaccines significantly reduced nasal colonization by *S. pneumoniae* strain D39 compared to immunization with DNA vaccines administered intramuscularly (i.m.). These findings are related to the high level of sIgA in the nasal washer, as well as systemic IgG antibodies and a shift toward a Th1-mediated immune response [64].

One of the main problems with DNA vaccines delivered by live *Salmonella* vaccines is that the DNA cannot effectively contact with the cytosol and then the nucleus of eukaryotic cells to initiate transcription and translation of encoded antigen genes. Besides those described above, other modifications could be used to increase the efficiency of DNA vectors delivered by *Salmonella*. Generally, there are two barriers for *Salmonella* delivering DNA vaccines into the cytosol. The first is that *Salmonella* resides in *Salmonella* containing vacuole (SCV) after entering the cell, which isolates *Salmonella* from other cytosolic components. This problem can be conquered by using a strain with the *sifA* mutation [102]. SifA is critical to maintain the SCV. Mutating *sifA* disrupts the vacuoles [103]. The second problem is the cell membrane/wall of *Salmonella*. This can be conquered by using a regulated delayed lysis *in vivo* strategy [46]. This strategy enables effective lysis of bacteria to release the bacterial cell components, including DNA vaccines. Combining these two approaches has led to promising results for a influenza vaccine [102]. 

#### 2.1.4. *S. typhi* Clinical Trial

Due to the lack of an animal model, progress to develop safe *S. typhi* vaccines for human use is slow. Currently, a clinical trial is still the best measurement of safety and effectiveness of *S. typhi* vaccines or vaccine vectors. Our intensive work carried out in mice lead to the development of a *S. typhimurium* strain χ9558 with a balance between safety and immunogenicity in adult, neonatal and infant mice [47,49,52,54,56,91,92]. Based on the results, we constructed three recombinant attenuated *S. typhi* vaccine vectors, χ9633, χ9639, χ9640, with essentially the same genotype as χ9558 carrying plasmid pYA4088 encoding the α-helical fragment of PspA Rx1 (aa 3–285) [65], but with an additional mutation eliminating the immunosuppressive capsular Vi antigen [65,66]. The vectors were constructed to test the hypothesis that the immunogenicity of live *Salmonella* vaccines is, at least in part, on its RpoS status. All three *S. typhi* vaccine strains are similar to the licensed live attenuated typhoid vaccine Ty21a in their abilities to survive in human blood and human monocytes. They are more sensitive to complement and less able to survive and persist in sewage and surface water than their wild-type counterparts [65]. Adult, infant and neonatal mice immunized with these vectors develop immune responses against PspA and *Salmonella* antigens. The percentages of protection against *S. pneumoniae* challenge in adult mice immunized with these vectors are between 50 and 81.3% [65,66]. In the pre-clinical setting, they achieved the desired balance between safety and immunogenicity in adult, neonatal and infant mice [65,66]. These strains were tested in a dose-escalation clinical trial from 10^7^ to 10^10^ CFU to further evaluate the safety and immunogenicity and determine which of the three *S. typhi* vectors has the optimal safety and immunogenicity profile in human hosts [40]. The results proved that the vaccines are safe and well tolerated. Even in the highest dose group, no subject experienced severe reactions or serious adverse events. The vaccine is also very safe to the environment without any shedding of viable vaccine cells in stools. This is a very important feature because bacteremia and shedding are not acceptable for the development of a vaccine for use in neonates/infants and for use in immunocompromised hosts, especially persons infected with HIV. However, only a limited number of subjects had increased levels of anti-PspA IgA. The inability to stimulate significant immune responses to PspA is not clear. It may relate to the high pre-immunization antibody titers against *S. typhi* and PspA likely due to previous *Salmonella* infection and pneumococcal vaccination, possible over-attenuation or limited in vivo slow growth of the attenuated *S. typhi* strains. In this last regard, the use of the regulated delayed synthesis of PspA *in vivo* might have been due to too much repression and insufficient cell divisions of the vaccine strains to adequately reduce repressor concentration by cell division. Based on the trial results, the vaccine strains have been further modified to increase protective antigen production and delivery to increase immune responses. An improved version of *S. typhi* based on the most promising vaccine strain χ9640 carrying vectors encoding multiple protective pneumococcal antigens is being developed and evaluated. 

#### 2.1.5. Issues

Human-restricted *S. typhi* is the choice for oral human vaccines because it can effectively invade mucosal tissues and enter systemic sites, leading to strong mucosal, humoral and cellular immune responses. *S. typhimurium* only causes self-limited gastroenteritis in human. It is less capable to invade beyond the gut mucosa in healthy humans and less able to stimulate long-lasting immunity [104]. Thus, it is not actively pursued as an oral human vaccine [105]. To evaluate the attenuation and immunogenicity of *S. typhi* vaccine strains, infection of mice with *S. typhimurium* is used as an experimental model because *S. typhimurium* infection in mice results in typhoid-like diseases in mice, which likens *S. typhi* infection in human. Although *Salmonella* shows promise as a vaccine vector and has been extensively tested, there is still no licensed RASV. In addition to the issue of immunogenicity, the key concern associated with RASVs is safety, especially in newborns/neonates, the elderly and those who are immunocompromised or have chronic diseases. Clinical results demonstrated that our RASVs χ9633, χ9639 and χ9640 are safe and non-shedding, but less immunogenic [40]. This is different from what we observed in mice with *S. typhimurium* as a model. Our *S. typhimurium* vaccine vector χ9558 carrying plasmid pYA4088 induced significant anti-PspA IgG/IgA antibody titers in mice, *S. typhi* vaccine strains χ9633, χ9639 and χ9640 with the same genotype carrying the same plasmid did not induce significant anti-PspA IgG/IgA responses in the clinical trial. The next step is to increase their immunogenicity while maintaining safety. Attaining the desired balance between safety and immunogenicity is difficult, especially for *S. typhi* vaccines, which lack a relevant animal model. To develop a *S. typhi* vaccine, evaluation of *S. typhimurium* strains of similar genotype and phenotype in mice is used as a close mimic of *S. typhi* in humans. Considering the differences between the human and mouse mucosal lymphoid system [106,107], several humanized immune system mouse models displaying classical manifestations of human typhoid fever including meningitis, liver pathology and mortality were developed [108,109,110,111]*.* However, they still have problems with variations in ability of *S. typhi* to attach to, invade into, survive intracellularly and distribute into internal effector lymphoid tissues. These disadvantages and the high cost of these humanized mice still limit their current use. Further research and improvement of these humanized mouse models should ultimately aid in developing a safe and effective *S. typhi* vaccine vectors for humans [108].

Currently, the mouse is still the most cost-effective model for testing safety and efficacy of RASVs. Most studies with *S. typhi* RASVs used adult mice, but a few studies have adopted using newborns and neonates in developing pneumococcal vaccines [52,66,91] (Table 1). We still lack safety and efficiency data in aged or in malnourished mice. Baby mice and aged mice have different T- and B-cell responses that affect induction of optimal immune responses to vaccines [112,113,114,115,116,117,118,119,120,121,122]. In newborn/neonate mice, the presence of maternal antibody enhanced immune responses and protections against *S. pneumoniae* challenge [52,66]. These responses include increased IgG, IgA and IL-4-secreting levels in mice immunized with *S. typhimurium* vaccine and enhance levels of mucosal IgA, IFN-γ, and IL-4 in mice immunized with *S. typhi* vaccines [52,66]. All these factors affect the performance of the vaccines. Considering that the newborn/neonatal and aged people are the main high-risk groups, more efforts should be put into using young and aged mice to evaluate the candidate RASV-*S. pneumoniae* vaccines. We may need age-specific RASVs for different age groups.

### 2.2. LAB Deliver Pneumococcal Antigens

#### 2.2.1. Benefit of LAB: Safety, Adjuvant and Prevention of *S. pneumoniae*

LAB are a group of Gram-positive bacteria that produce a common end product—lactic acid—from the fermentation of sugars [123]. These non-sporulating bacteria include species of *Lactobacillus*, *Lactococcus*, *Leuconostoc*, *Pediococcus* and *Streptococcus* [123]. Due to limited biosynthetic abilities for pre-formed amino acids, B vitamins, purine and pyrimidine, their habitats are restricted to the place, such as intestine, where the required compounds are abundant [123]. They have positive effects on human and animal health [124,125] and were widely used for food without causing any known health problem for thousands of years. This status is considered GRAS (generally recognized as safe). 

LAB can be used as adjuvants for their immunostimulatory properties [126,127,128,129,130]. This GRAS status of LAB in adults and infants and their abilities to stimulate immune responses make them very attractive candidates for the development of mucosal vaccines [131]. Most LAB induce Th1 type responses, some LAB can induce different arms of the immune response [132], like *L. reuteri* induces Th2 responses and *L. rhamnosus* induces Th17 responses [126,129]. *Lactobacillus* and *Lactococcus* are the main vaccine vehicles to delivery heterologous proteins or DNA to mucosal tissues (see reviews [123,133,134,135]). 

Live LAB have been shown to be effective adjuvants to improve the immune responses against respiratory pathogens [75,136]. *In vitro*, *L. rhamnosus* can inhibit *S. pneumoniae* adherence to human epithelial cells [137]. Ingestion of LAB reduces nasal colonization by *S. pneumoniae* in humans [138]. Oral administration of *L. lactis* in mice can improve clearance of pathogens from the lungs, reduce lung injuries, and increase survival of mice against *S. pneumoniae* infection [139,140]. The mechanism is related to an up-regulation of the respiratory innate and specific immune responses, like improved production of TNF-α in bronchoalveolar lavage (BAL) fluid, enhanced recruitment of neutrophils into the alveolar spaces, increased activation of BAL phagocytes, and improved production of BAL IL-4 and IL-10 [141]. These responses stimulate the IgA cycle, increase IgA^+^ cells in the intestine and bronchus, and increase BAL anti-pneumococcal IgA and IgG levels [141]. Nasal administration of *L. fermentum* in mice can increase protective responses against *S. pneumoniae* challenge by stimulation of neutrophil activity or by the increase of the number of activated macrophages and lymphocyte populations in the tracheal lamina propria [142,143]. Nasal administration of *L. lactis* improves local and systemic immune responses against *S. pneumoniae* with reduced nasal colonization, increased clearance rate of *S. pneumoniae* from lungs, reduced dissemination of pneumococci into blood and reduced damage to respiratory tissues, which is also related to the up-regulation of the innate and adaptive immune responses in both local and systemic compartments as well as different cytokine responses [143,144]. These responses increase the pulmonary lymphocyte population, anti-pneumococcal IgA and IgG in bronchoalveolar lavage (BAL) and serum, and phagocyte activation in lungs, blood and bone marrow [143]. Increasing resistance to pneumococcal respiratory infection was shown in both normal [140] and malnourished mice fed with *L. casei* [139]. However, the ability to induce these responses is varied among LAB species. Thus, different LAB strains are evaluated as vaccines or vaccine vectors delivering pneumococcal antigens against *S. pneumoniae* [67,76,131,145,146].

#### 2.2.2. LAB System: Promoters and Strains

Several pneumococcal candidate antigens, PsaA, PspA, PspC and PppA delivered by LAB, most in *L. lactis* NZ9000 or its parent MG1363, have been tested against *S. pneumoniae* challenge in animal models. The strain NZ9000 is derived from the strain MG1363 with the *nisRK* genes integrated into the *pepN* gene, which facilitate the use of the NIsin-Controlled gene Expression system-NICE [147,148]. Two strategies were adopted to develop LAB vaccines against *S. pneumoniae*: recombinant lactic acid bacterial vectors and non-genetically modified Gram-positive enhancer matrix (GEM) particles. We will focus on the live vaccine strategy. The GEM approach was discussed elsewhere [149,150,151,152].

To induce immune responses, especially antibody responses, higher antigen production is preferred. Thus strong promoters are adopted. Although several promoters were used in LAB [133], two promoters were used more in LAB-*S. pneumoniae* vaccines. One is the lactococcal promoter P1 and another is the nisin-regulated promoter [69,71,153]. The P1 promoter, which was used to express *psaA*, *pspA* and *pspC* [69,71,153], is a constitutive promoter. It is a medium strong promoter from the *L. lactis* genome [154]. However, continuous high-level production of heterologous proteins could result in intracellular accumulation, aggregation and degradation of proteins in the cytoplasm and lead to deleterious effects to the cells [134]. To solve this problem, two methods were adopted. One way was to use a system that can regulate protein synthesis. The most widely used system is the NICE system. 

The nisin-regulated promoter system has several advantages. The transcription of the promoter P_nis_ can be efficiently controlled by the extracellular concentration of the antimicrobial peptide nisin through the two-component regulatory system, sensor NisK and regulator NisR [148,155,156], which provides a simple way to control gene expression. The benefits of this system are: the small size of the promoter, which can be trimmed down to less than 50 bp; hyper-production of protein, which can reach up to 50% of the total protein; tightly controlled gene expression with undetectable protein synthesis without induction; very high dynamic induction range to 1,000-fold dependent on the concentration of nisin and can be used in a variety of LAB [147,157]. For maximum induction, the nisin concentration is 10 ng/mL (3 nM), which is the MIC (minimum inhibitory concentration) value for nisin [148]. As an antimicrobial peptide, nisin can repress the growth of Gram-positive bacteria and is regarded as a food-grade preserver. In strains, like *L. lactis* F17847, with the NICE system, the induction of antigen synthesis with nisin before immunization is necessary. Other strains, like *L. lactis* NZ9700, can produce nisin to omit this process. However, it also means that the NICE system is converted into a non-regulated system. Basically, the NICE system is a system that regulates protein synthesis *in vitro*, not *in vivo*. 

Another way to reduce the metabolic burden on the LAB vector is to secret the protein into the periplasm, onto the cell wall or into the supernatant. The secreted protein can directly interact with the environment. Several protein secretion systems in LAB have been discussed [133,134,158]. Currently, the Usp45 signal was most frequently adopted. Usp45 is the most abundant protein secreted by *L. lactis*. The secretion of Usp45 is through the Sec pathway. Adding negative charge peptides at the *N*-terminal part of the mature moiety will improve the translocation efficiency across the cytoplasmic membrane [159,160]. Thus, this secretion signal is widely used to deliver heterologous antigens [69,153]. However, some researchers do not use this secretion protein. The reason may lie in the fact that location of the protective antigen protein is not important for induction of the immune response by LAB. Thus, for LAB-delivered antigens, the amount of antigen produced is more important than the location of the antigen, especially when delivered by mucosal routes, i.n., intragastric, and oral routes [161,162,163]. This is in contrast to *Salmonella*, in which periplasmic secreted antigen induced higher antibody responses than cytoplasmic antigen [44]. Although the surface synthesis protein may increase its presentation to the immune cells, it is also prone to be proteolytic degraded extracellularly or denatured by the acid or bile in the gastrointestinal tract in oral vaccinations [158].

Recombinant LAB delivering pneumococcal antigens is mainly by the i.n. route. LAB strains were used to deliver PsaA, PspA and PspC by nasal immunization [67,69,153] and PppA by oral route [73]. In these reports, the plasmid-based antigen gene expression system was used. Until now, there are no reports using chromosomal-based antigen gene expression system in LAB for *S. pneumoniae* vaccines. 

#### 2.2.3. Antigens

##### 2.2.3.1. PsaA

Mice intranasally immunized with some species of *Lactobacillus* synthesizing PsaA developed systemic anti-PsaA IgG and IgA responses and displayed reduced pneumococcal colonization upon nasal challenge [67]. The immune responses depended on the amount of PsaA production, which vary in *Lactobacillus* from 20 to 250 ng/10^9^ cells [67]. *L. plantarum* and *L. helveticus* induce significant IgA responses in nasal and bronchial washes and IgG in serum as well as reduced nasal colonization of *S. pneumoniae* 6B compared with the saline control group. However, when compared with strains carrying the control vector, only the recombinant *L. helveticus* led to a significant reduction of pneumococcal nasal colonization. Although *L. casei* does not generate significant antibody responses, it results in reduced colonization compared with the saline group, but not the vector control. These results reflect that LAB strains have different adjuvant properties. The three LAB strains synthesize similar amounts of PsaA (150–250 ng/10^9^ cells). They persist in the mice nasopharynx after inoculation for three days except *L. casei* [67]. Short persistence with the low level of antigen production is not enough for the stimulation of antibody responses in nasal and systemic sites. Thus, using LAB for a vaccine needs to consider the protein synthesis levels, persistence and intrinsic adjuvant properties of different LAB [67]. 

##### 2.2.3.2. PspA

*L. casei* and *L. lactis* were used to deliver PspAs. *L. casei* delivered PspAs from clades 1 and 5 under the control of the constitutive P1 promoter. The PspA synthesized retains in the cytosol [69,70]. Mice intranasally immunized with *L. casei*-PspA1/5 develop significant anti-PspA IgG, but no IgA in nasal washes, saliva or vaginal washes [69,70]. Previous experiments showed that PspA antigen is not effective for inducing IgA without adjuvant [164,165], thus *L. casei* seems not to display adequate adjuvant activity [69]. The anti-PspA1 antibody can effectively bind to PspA clade 1 and clade 2 and induce different amounts of complement deposition on the pneumococcal surface depending on the serotypes and PspA clades of *S. pneumoniae*. Mice immunized with *L. casei*-PspA1 show increased survival times when compared to mice immunized with saline against lethal i.p. pneumococcal challenge [69]. However, the percentage of protection against i.p. challenge was only 33.3% although the mice were immunized six times [69]. *L. casei*-PspA1 bacteria can be recovered up to five days after the i.n. inoculation with 10^9^ CFU on two consecutive days. The presence of PspA antigen does not affect the ability of strain colonization in the nasal pharynx [69]. Another construction with *L. casei* delivering PspA5 conferred protection against i.n. pneumococcal challenges in mice. This was accompanied by the increased secretion of IFN-γ by lung cells against invasive pneumococcal challenge [70]. 

Intranasal immunization with *L. lactis* with intracellularly produced PspA using the NICE system induced not only serum anti-PspA IgG, but also lung lavage anti-PspA IgA [68]. This result further strengthens the conclusion that LAB strains have different adjuvant activities. Immunization with *L. lactis*-PspA significantly protected mice against i.p. challenge with the *S. pneumoniae* TIGR4 strain than protein administered intranasally or control groups did. The protection induced by *L. lactis*-PspA is similar to that induced by PspA/Alum administered by the subcutaneous (s.c) route. This was attributed to the Th1-mediated immune responses induced. In an intranasal challenge model, *L. lactis*-PspA afforded the highest protection among the levels elicited with purified PspA administrated i.n. or s.c. with adjuvant or in control groups [68]. About 20% of control mice survive the challenge suggesting that *L. lactis* may contribute to non-specific host immunity.

##### 2.2.3.3. PspC

Ferreira *et al.* reported that *L. casei*-PspC (from CPS type 6B) without an SS cannot confer significant protection against i.n. pneumococcal challenge although it induces IFN-γ secretion in lung cells and IL-17 secretion in both lung and spleen cells [70]. This may relate to the low homology of the PspC amino acid sequence between the vaccine (CPS type 6B) and the challenge strain (CPS type 3). Further, Hernani *et al.* tested *L. casei*-PspC with or without an SS using different immunization protocols, intranasal, sublingual and primer-boosting with PspC protein. However, none of these protocols induced significant levels of anti-PspC antibodies in vaginal or nasal washes and serum before challenge [71]. Despite these results, nasal immunization of mice with *L. casei*-PspC without a SS significantly reduced pneumococcal colonization by strain 0603 with an increase of anti-PspC IgA in the nasopharynx five days after challenge [71]. *L. casei* carrying cell-wall-associated PspC only marginally reduced pneumococcal colonization after challenge. Thus, the reduced colonization of *S. pneumoniae* may be attributed to the non-specific adjuvanticity of *L. casei*. These results show that protection is only achieved by using a PspC with high identity at the *N*-terminal region to the PspC expressed by the *L. casei* vaccine strain. Considering the polymorphism of PspC [94], using different PspC molecular types to cover more pneumococcal strains will be necessary [71]. 

##### 2.2.3.4. PppA

An antigenically conserved antigen, PppA [166], delivered by *L. lactis* NZ9000 on the cell surface, was tested as live or inactive vaccines using intranasal and oral routes in adult and young mice [72,73,74,75]. Nasal and oral immunizations of *L. lactis*-PppA induced anti-PppA IgM, IgG and IgA responses in serum and bronchoalveolar lavage fluid in both adult and young mice. The responses are significantly higher in young mice than in adult mice. The challenge results showed that intranasal immunization with *L. lactis*-PppA could confer protection against homologous *S. pneumoniae* i.p. challenge by either active immunization or passively by antibody from immunized mice and increased resistance to respiratory infection with different pneumococcal serotypes (3, 6B, 14, 23F) in young and adult mice [72,73,74,75]. Oral immunization with *L. lactis-*PppA provided cross-protective immunity against four CPS types of pneumococcal strains with reduced lung bacterial counts [73]. Passive protection experiments proved that antibody is critical for protection [72]. There are no antibodies against *L. lactis* found in the serum or the BAL fluid in adult and young mice immunized with the recombinant strain. Thus, the host immune responses are directed against the protein expressed by *L. lactis*, not the vector [72]. These researches also show that vaccination at an early age of mice with the *L. lactis*-PppA strain is more effective [72]. 

##### 2.2.3.5. CPS Antigens

LAB were also used to deliver type 3 or type 14 CPS with the *eps* natural promoter to express the *eps* genes or the nisin-induced promoter to express the regulatory genes, respectively [76,77]. The CPS synthesized in LAB either associated with the cells (type 3 CPS) [76] or in the supernatant (type 14 CPS) [77]. The mice immunized with *L. lactis* expressing 0.5 μg of type 3 CPS or 0.5 μg of purified type 3 CPS from pneumococcus elicited similar titers of T-cell-independent anti-CPS IgM and IgG antibodies in the serum [76]. The LAB did not affect the T-cell-independent nature of the anti-CPS antibody responses [76]. Thus, *L. lactis* is a potential host for capsular synthesis. However, there is no animal protection study with CPS delivered by LAB vectors. 

#### 2.2.4. Issues

##### 2.2.4.1. Multi-dose and Immunization Route

Though LAB are normally ingested orally, most work with LAB delivering *S. pneumoniae* antigens used i.n. immunization (Table 1). One of the reasons is that LAB are non-invasive bacteria, which implies that antigen delivery to antigen-presenting cells is not as effective as when using invasive attenuated pathogenic bacteria. Intranasal vaccination with recombinant LAB can elicit protective immunity in both mucosal and the systemic compartments [167,168]. To avoid mucosal tolerance, administration of high dose (10^8^) of LAB for consecutive five days is preferred to induce IgA responses [73,141]. Most reports use three or more immunizations (Table 1). This increases the costs of the vaccines. Though LAB have been tested in clinical trials as food supplements/adjuvants for several vaccines, such as rotavirus vaccine, oral polio virus vaccine, influenza vaccine and oral cholera vaccine [169,170,171], it is unknown if the necessary doses to use for humans will be feasible and cost effective [131]. Also, for the nisin induction system, pre-induction and extensive washing to remove nisin are required before immunization. Thus, the complex immunization procedure should be addressed with the development of a new protein synthesis system and better procedures for preparing the LAB vaccines. 

Another issue related to the LAB vaccine is the safety concerns of i.n. immunization. I.n. immunization of mice with recombinant LAB induces excellent immune responses to the expressed antigens. Since the cribriform plate is a thin, well-hidden bone in the nasal cavity with numerous perforations for allowing passage of the olfactory nerves to the brain, there exists a potential route for bacteria to enter the cranial cavity if administered by i.n. route. Immunization with *Salmonella* by i.n. route results in brain colonization if the bacteria is not fully attenuated [92]. The GRAS feature of LAB is mainly based on the oral route. Thus, it would be helpful to check whether there is a problem using i.n. immunization with LAB-vectored vaccines. 

##### 2.2.4.2. Antibiotic Selection

Another disadvantage with the LAB-*S. pneumoniae* vaccines is the use of antibiotic-resistance markers, which are considered unacceptable in live vaccines due to the potential for antibiotics in the final product and the possible contamination of the environment with recombinant drug-resistant bacterial strains. The regulatory agencies also prohibit the usage of antibiotics in vaccine formula. Two antibiotics are commonly used in the LAB vectors. The first one is erythromycin. Erythromycin can inhibit the protein synthesis by binding the 50s subunit of the bacterial 70s rRNA complex. Most plasmids used in LAB vaccines have an erythromycin-resistance selection marker. This antibiotic is necessary to select the recombinant plasmid. LAB strains with recombinant plasmid are grown with erythromycin prior to immunization to maintain the plasmid. Adding the antibiotics not only increases the costs of the final product, but also raises the concern about the plasmid stability. The most used pTREX vectors have poor segregational stability in the absence of antibiotic selection [172]. LAB could lose the recombinant plasmid *in vivo* and lead to compromised immune responses. To conquer this problem, the use of the balanced-lethal strategy could be attempted with LAB vaccines. Another is the nisin. Nisin is a polycyclic lantibiotic produced by *L. lactis* to eliminate other competing Gram-positive bacteria. It is commonly used as a safe food preservative against bacteria, yeast, and molds. Nisin can bind to lipid, dissipate the membrane potential, induce efflux of cytoplasmic components and inhibit bacterial cell growth [173]. It is used as the inducer for LAB strains with the NICE regulatory system. Induction with nisin and extensive washing is thus required for synthesis of the antigens before inoculation into the immunized hosts [72], which adds complexity to the production process and increases the costs. Both erythromycin and nisin are broad-spectrum antibiotics against many bacteria. Although nisin is not a big problem for the LAB vaccines, the use of erythromycin raises concerns that this antibiotic may interfere with the normal flora in the human intestinal tract or nasopharynx. It was reported that the erythromycin-resistance gene can easily transfer from LAB to *Listeria* spp. at a frequency as high as 10^–4^ [174]. Nisin can also exert some immunomodulatory effects at high concentration [175]. Therefore, LAB vaccine with biocontainment properties to prevent their spreading of heterologous DNA is necessary. To conquer this problem, auxotrophic bacterial strains complemented by a wild-type gene in a cloning or expression vector was developed, such as the purine, threonine, pyrimidine, thymidine and alanine auxotroph [176,177,178,179,180]. Although these balanced-lethal systems were developed for the food industry, and their application in vaccine research have not been reported, they paved the way for their application in vaccine research. 

##### 2.2.4.3. Selection of Different LAB

*Salmonella* need to be attenuated to achieve a balance between safety and immunogenicity for vaccine application. It is not necessary to generate and evaluate mutations in LAB due to their GRAS feature. However, LAB have different abilities to modulate the immune system, the careful selection of LAB should be noted as a key factor that influences the results. Different LAB strains induce distinct cytokine profiles and exert different effects on the immune system [126,181,182,183]. Immunostimulating properties of LAB have been proved to be strain-, dose-, and even growth-phase-dependent [139,140,183,184]. Only *L. casei* CRI 431, *L. lactis* NZ9000 and *L. rhamnosus* CRL1505 have proved to be able to increase the resistance of mice to challenge with respiratory pathogens. A human study showed that *L. rhamnosus* GG has different immune modulation functions [185]. It stimulates immune functions in healthy persons, but down-regulated an inflammatory response in allergic persons. An oral *S. typhi* vaccine administrated with *L. rhamnosus* GG induced higher numbers of IgA-secreting cells, while with *L. lactis* induced higher numbers of CR3 receptor expression on neutrophils [183]. *L. lactis*-PspA can induce effective IgA responses, but *L. casei-*PspA is poor for induction of an IgA response [68]. Although the PsaA is synthesized on the surface of different LAB, including *L. lactis*, *L. casei*, *L. plantarum* and *L. helveticus* (Table 1), only lactobacilli lead to a decreased pneumococcal recovery from the nasopharynx upon a colonization challenge, but not *L. lactis* due to its low level of PsaA synthesis, which is not enough for inducing adequate humoral responses [67]. Thus, the selection of LAB should consider the intrinsic properties and the appropriate doses of each LAB. Optimum dose, frequency and duration of treatment for using LAB vaccines should be carefully compared and demonstrated through rigorously designed studies. 

### 2.3. BCG Delivers PspA

Bacillus Calmette-Guérin (BCG), a live attenuated strain of *Mycobacterium bovis*, was used as an effective vaccine for *M. tuberculosis*. It has been given to 3 billion people worldwide since 1948, with a very low incidence of serious complications, even for young children and infants [186]. Besides the common benefits as a bacterial vector [186], it has the immunostimulatory properties that can augment the immune responses against routine immunizations in infant [187]. This live attenuated vaccine establishes a persistent infection and induces both cellular and humoral immune responses. Currently, BCG is shown effective in preventing the several forms of TB in toddlers, which may be a benefit for delivering pneumococcal antigen for newborns and infants since they are the main target population to prevent *S. pneumoniae* infections. BCG was used to deliver PspA antigen in the cytoplasm, associated with cell membrane or in a secreted form. Although the peak antibody titers elicited by BCG expression *pspA* with or without a secretion signal did not differ markedly, protective responses were observed only in mice immunized with BCG expressing *pspA* with its native signal peptide, which leads to the exportation of PspA, or as a fusion with the Mtb19 lipoprotein signal peptide, which results in it being anchored to the cell membrane. These results were observed in both BALB/c and C3H/HeJ mice using an i.p. challenge model [78]. The antiserum can also passively protect CBA/B (Xid) mice, which are highly sensitive to *S. pneumoniae* challenge [188], against other *S. pneumoniae* virulent strains exhibiting heterologous PspAs and CPS types [78]. Thus, the BCG-PspA is another potential live vaccine for inducing humoral immune responses against pneumococcal infections. However, the induction of cellular immune responses were not addressed in this report. Recently, progress in rBCG research may pave the road for further use of BCG as an effective vaccine vector for *S. pneumoniae* [189]. 

## 3. Viral Vector Delivers Pneumococcal Antigens

There are a few reports using viral vectors to deliver pneumococcal antigens. Arévalo *et al.* used replication-defective recombinant adenoviruses Ad5 (rAds) to deliver PspA, PsaA and PdB, either individual or combined [79]. rAds can direct high levels of viral gene expression in mammalian cells and induce strong immune responses [190]. The rAds used here cannot replicate in the host due to the lack of the packaging elements [191]. The results show that mice intranasally immunized with rAds carrying each of the three antigens develop robust antigen-specific serum IgG responses. Mice immunized with rAds carrying three antigens develop slightly reduced antibody responses against PspA, PdB and PsaA compared with the mice immunized with rAd carrying the individual antigen at 6 and 10 weeks. Two-dose vaccination induced stronger antibody responses, but cannot increase them further by a third boosting. rAd/PdB alone does not reduce the lung colonization carriage. Both rAd/PdB+rAd/PsaA or rAd/PdB+rAd/PspA can lead to reduced lung colonization of *S. pneumoniae*. rAd/PdB+rAd/PspA+ rAd/PsaA is most effective in reducing the bacterial load in the lung after challenge. 

## 4. Conclusions

Protein-based vaccines are the future for *S. pneumoniae* vaccine research [26]. However, protein vaccines have problems of high costs related to their complex manufacturing process. Iyer *et al.* reported that candidate antigens, such as PsaA, Stkp, PcsB, show different requirements for stability when combined with different adjuvants and excipients [192]. PsaA needs sodium phosphate to be stable when it absorbs to Alhydrogel, but StkP does not need. Thus, for a multi-antigen vaccine, separate storage of each protein for long-term storage stability might be necessary, leading to further cost increases for a mixed protein vaccine. Recently, a fast-dissolving tablet formulation of a live attenuated enterotoxigenic *E. coli* was developed [193]. The tablets rapidly disintegrate *in vivo* but preserve the bacteria at 2–8 °C for at least 12 months with only 0.4 log_10_ loss of viability during storage. These results provide a practical option for formulating ETEC vaccines or other live bacterial vaccines for oral immunization and help to facilitate delivery of lifesaving vaccines, particularly in low-resource settings [193]. Our experiments showed that RASV vaccine strains stocked at −80 °C do not change their titers after storage for five years [194]. Whether there is a change in the immunogenicity still needs to be probed. These results along with other attributes discussed above demonstrate the cost benefits of using recombinant live vector technologies. 

*S. pneumoniae* causing disease involves multiple steps, including attaching to, invading into and spreading in the host [27]. An effective vaccine could block any one of these steps, but preferably all of them. *S. pneumoniae* also have over 90 serotypes with different pathogenic attributes. The vaccine should protect against infections by most of the serotypes. The ideal vaccine should include multiple antigens to stop multiple steps of infection and confer protection against multiple serotypes. In the future, work should focus on delivering multiple antigens and test protection with multiple challenge models against multiple serotypes. Currently, the challenge studies in evaluating recombinant live vaccine vectors used serotypes 2, 3, 4, 6B, 14, and 23F (Table 1), which did not include some serotypes, like 1, 5, 6A, 7F, 9V, 18C, 19A, 19F in the 13-valent conjugate vaccine. An effective vaccine needs to protect against all these serotypes. The inclusion of multiple antigens protecting against *S. pneumoniae* strains in multiple families with regard to the PspA and PspC antigens is therefore important. The inclusion of conserved antigens such as PsaA and Ply and the conserved proline-rich domains of PspA and PspC should further augment the protective efficacy of these vectored vaccines [62,63].

Much progress has been made in improving the technologies to design, construct and evaluate live bacterial vectored vaccines against *S. pneumoniae*. These improvements enhance safety, tolerability and effectiveness in inducing protective immunity. Three *S. typhi* vaccines based on these technologies have been tested in a phase I clinical trial. As for LAB based vaccines, there is still room for improvement. In the future, combining the benefits of LAB and *Salmonella* will be possible. With the advancement of knowledge about bacteria—not only *Salmonella* and LAB, but also other bacteria interacting with hosts—and by using new technologies, we should finally be able to develop safe, efficient, and relatively inexpensive needle-free vaccines against *S. pneumoniae,* as well as other pathogens. 
